# Lung Injury Induced by TiO_2_ Nanoparticles Depends on Their Structural Features: Size, Shape, Crystal Phases, and Surface Coating

**DOI:** 10.3390/ijms151222258

**Published:** 2014-12-03

**Authors:** Jiangxue Wang, Yubo Fan

**Affiliations:** Key Laboratory for Biomechanics and Mechanobiology of Ministry of Education, School of Biological Science and Medical Engineering, Beihang University, Beijing 100191, China; E-Mail: wangjiangxue@buaa.edu.cn

**Keywords:** TiO_2_ nanoparticles, nanotoxicology, physicochemical property, lung injury, pulmonary inflammation

## Abstract

With the rapid development of nanotechnology, a variety of engineered nanoparticles (NPs) are being produced. Nanotoxicology has become a hot topic in many fields, as researchers attempt to elucidate the potential adverse health effects of NPs. The biological activity of NPs strongly depends on physicochemical parameters but these are not routinely considered in toxicity screening, such as dose metrics. In this work, nanoscale titanium dioxide (TiO_2_), one of the most commonly produced and widely used NPs, is put forth as a representative. The correlation between the lung toxicity and pulmonary cell impairment related to TiO_2_ NPs and its unusual structural features, including size, shape, crystal phases, and surface coating, is reviewed in detail. The reactive oxygen species (ROS) production in pulmonary inflammation in response to the properties of TiO_2_ NPs is also briefly described. To fully understand the potential biological effects of NPs in toxicity screening, we highly recommend that the size, crystal phase, dispersion and agglomeration status, surface coating, and chemical composition should be most appropriately characterized.

## 1. Introduction

Owing to the rapid development of nanoscience and nanotechnology, many kinds of engineered nanoparticles (NPs) are needed and produced. It has been reported that there are more than 2800 nanoparticulate-based applications commercially available in various areas, such as electronics, materials, medicine and energy [1]. A new generation of materials, nanomaterials/nanoparticles are widely used in our daily life in water treatment, clothing, food additives, implants, and disease diagnosis, *etc.* Therefore, new issues are raised by researchers and scientists regarding the potential health effects and environmental impact of NPs [2,3,4,5].

Nanoscale titanium dioxide (TiO_2_, diameter < 100 nm), one of the most commonly manufactured NPs, is a noncombustible and odorless white powder that is employed as a white pigment in paints and papers, a photocatalyst in solar cells, an optical coating in ceramics, and a corrosion-protective coating in bone implants, *etc*. It naturally exists in three crystal structures: anatase (tetragonal), rutile (tetragonal), and brookite (orthorhombic). Anatase and rutile TiO_2_ both have a tetragonal structure, while the TiO_6_ octahedron of anatase TiO_2_ is distorted to be larger than that of the rutile phase [6]. Rutile TiO_2_ is stable at most temperatures, while anatase is not at an equilibrium phase and is kinetically stabilized. At temperatures between 550 and 1000 °C, anatase transforms to the equilibrium rutile phase. Brookite TiO_2_ is formed with the edge-sharing TiO_6_ octahedron and has a larger cell volume. This form of TiO_2_ is not often used in research. TiO_2_ has a very low dissociation constant in water and aqueous systems, thus, it is insoluble in water and organic solvents, as well as under physiological conditions.

Generally, TiO_2_ is considered a poorly soluble, low toxicity NP [7,8,9]. However, Ferin *et al.* observed that ultrafine TiO_2_ (~20 nm) accessed the pulmonary interstitium. The acute inflammatory response was indicated in this study by polymorphonuclear (PMN) leukocytes among lavaged cells in rat lung after acute instillation and subchronic inhalation [10]. Oesch and Landsiedel [11] reviewed the genotoxicity of nanomaterials, including nanosized TiO_2_, which varied in the test systems used. Positive and negative results were obtained in the DNA damage and gene/chromosome mutation tests. When different sizes of the same form of TiO_2_ were tested in the same laboratory, smaller material induced DNA damage and micronuclei formation while large size material did not [11].

Among the several routes of nanosized TiO_2_ exposure, inhalation is apparently a more general and important route of exposure to NPs than others like injection, ingestion, and dermal penetration. A few epidemiologic studies have surveyed the carcinogenicity of TiO_2_ in workers employed in TiO_2_ production factories by considering the pathophysiology, gender, age and exposure pathways, which were reviewed by NIOSH in 2005 [12]. There is little clear evidence of elevated risks of lung cancer mortality or morbidity among workers exposed to TiO_2_ dust [13,14,15]. In 2006, the International Agency for Research on Cancer (IARC) classified pigment-grade TiO_2_ as “possibly carcinogenic to human beings (Group 2B)”, based on the carcinogen policy of the Occupational Safety and Health Administration (OSHA), according to sufficient evidence of carcinogenicity in animals and inadequate evidence for human carcinogenicity [16]. 

The lung is a primary target organ of NPs exposure via inhalation in the occupational setting. The spectrum of the toxic effects of nanoscale TiO_2_ on pulmonary responses has raised much concern. When searching for “nano TiO_2_ and pulmonary” in PubMed, there are 531 results, and of those results, over one-half (304 results) has been published in the last 10 years, and approximately 40% (nearly 207 papers) have been published in the last five years. An increased incidence of lung injury and pulmonary inflammation induced by exposure to TiO_2_ NPs has been reported in the scientific literature. Sub-chronic and chronic (inhalation or intratracheal instillation) studies have revealed that TiO_2_ NPs are deposited in the lung and translocated to the lymph nodes [5,17,18,19]. The overload of TiO_2_ NPs in the lung can exceed the ability of the macrophages to phagocytose and, eventually, transfer across the epithelium and migrate to the deeper pulmonary interstitium to induce the pulmonary inflammatory response in a dose-dependent manner. The most commonly reported biomarkers are largely detected by analyzing the bronchoalveolar lavage fluid (BALF) propertied and the pathology of the lung *in vivo*. An increased number of macrophages and neutrophils, fibroproliferative lesions and epithelial hypertrophy and hyperplasia in lung alveoli have been observed in exposed animals [20,21,22,23,24,25,26]. The clearing of particles from the alveolar region is much slower and may take weeks to years. Rats, mice and hamsters show different lung burden and clearance patterns for TiO_2_ NPs, and hamsters are better able to clear TiO_2_ NPs than similarly exposed mice and rats [14,17,18]. The pulmonary toxicity and tissue injury elicited by TiO_2_ NPs is based on several physical and chemical properties, which have been reported in many studies [27,28,29,30].

Dose is traditionally considered in toxicity screening. Paracelsus noted, “The right dose differentiates a poison and a remedy.” However, the interaction of TiO_2_ NPs with a biological system is closely correlated with their structural features including size, shape, crystal phase, and surface coating. In this paper, we will concentrate systematically on the influence of physicochemical features of TiO_2_ NPs on lung toxicity and pulmonary cell impairment. Although many related studies have been published recently, only selected representative works are cited here. The aim is to understand the correlation of the physicochemical properties of TiO_2_ NPs with their potential hazardous effects on lung tissue and to help improve their application performance.

## 2. Structural Features of TiO_2_ Nanoparticles

### 2.1. Size

Size is a key factor for nanoparticles. Based on an agreement on the size of nanoparticles among the groups of standards (International Organization for Standardization (ISO), American Society for Testing and Materials (ASTM), and Scientific Committee on Emerging and Newly Identified Health Risks (SCENIHR)), the scale from 1 to 100 nm defines the size range of a nanoparticle [31]. The properties of particles change as the size approaches nanoscale. The percentage of atoms becomes significant at the surface of NPs, and there are many active sites on the particle surface. Therefore, many researchers in “nanotoxicology” aim to elucidate the interaction of nanoparticles with biological systems and the mechanism(s) by which they act. For an overview, the lung toxicological effect in animal models and cytotoxicity of TiO_2_ NPs with different structural features were listed in Table 1 and Table 2. In 1992, Ferin *et al.* [10] first reported that the ultrafine TiO_2_ particles (~20 nm) were translocated to the lung interstitium to a greater extent and cleared from the lungs more slowly than fine TiO_2_ particles (~250 nm) in Fischer 344 rats after the intratracheal instillation of 500 μg of TiO_2_. Based on a predictive mathematical model (ICRP 1994) [32], Obserdorster *et al.* [5] figured out the fractional deposition of inhaled particles in different regions of the human respiratory tract, including three regions—the extrathoracic (mouth or nose and throat), the trachea-bronchial and the alveolar regions. NPs mainly deposit in the alveolar region, with approximately 50% of the 20-nm particles depositing efficiently in the alveolar region and only ~15% of this particle size depositing in tracheobronchial and nasopharyngeal regions. This model also supports another report that TiO_2_ particles (~20 nm) highly accessed the lung interstitium of the rat more than fine TiO_2_ particles (less than 200 nm) at an equal mass dose [20], resulting in the influx of PMNs into the alveolar space and a large acute pulmonary inflammatory reaction in BALF.

Alveolar macrophages (AM), as phagocytic cells, reside on the alveolar epithelium and clear solid particles through phagocytosis. The macrophage ability of AM has been compared in commercial ultrafine and fine TiO_2_ particles both *in vitro* and *in vivo*. Renwick *et al.* [33] reported that the ultrafine TiO_2_ (29 nm mean diameter, 50 m^2^/g surface area) significantly reduced the ability of J774.2 mouse AM to phagocytose 2 μm indicator latex beads more than the fine TiO_2_ (250 nm mean diameter, 6.6 m^2^/g surface area). Oberdörster *et al.* [34] compared the AM-mediated clearance of the same mass of ultrafine TiO_2_ (20 nm) and fine TiO_2_ (250 nm) in the rat. They reported that a volumetric loading of 9% with fine TiO_2_ caused a retention half-time of 117 days, whereas a volumetric loading of 2.6% with ultrafine TiO_2_ caused a prolongation of the clearance half-time (541 days). Gibbs-Flournoy *et al.* [35] also detected that 27-nm TiO_2_ was internalized into human bronchial epithelial (BEAS-2B) cells and proximity to cellular nuclei with time using darkfield and confocal laser scanning microscopy (DF-CLSM). For any given mass of particles, as particle size decreases, the total particle surface area increases dramatically [5]. The larger particle the surface area is, the greater the toxicity that the ultrafine particles will develop [20]. For the 29 and 250 nm TiO_2_, the specific surface area is 50 and 6.6 m^2^/g, respectively. After receiving 0.1 mg of nanoscale TiO_2_ (rutile, 21-nm average particle size; specific surface area of 50 m^2^/g), the lungs of ICR mice showed significant changes in morphology and histology, including the disruption of alveolar septa and alveolar enlargement (indicating emphysema), type II pneumocyte proliferation, increased alveolar epithelial thickness, and an accumulation of particle-laden AM [36]. On the contrary, after instillation with 1 mg fine TiO_2_ (250 nm mean diameter, specific surface area of 6.6 m^2^/g), the mice showed no inflammatory cells or expression of inflammatory cytokines in the lung tissue at 4, 24, or 72 h [37]. Rats treated with ultrafine TiO_2_ (29 nm), but not fine TiO_2_ (250 nm), by instillation had an increased percentage of neutrophils, γ-glutamyl transpeptidase concentration (a measure of cell damage), protein concentration (a measure of epithelium permeability), and lactate dehydrogenase (LDH) in BALF [33]. The genotoxicology of nanosized TiO_2_ particles on human bronchial epithelial cells was investigated in a size-dependent manner. Nanosized TiO_2_ particles (10 and 20 nm) induced the oxidative DNA damage by the strand breaks and base damage in the absence of light, but larger sized TiO_2_ (>200 nm) did not induce any DNA damaging events [38].

**Table 1:** Lung toxicity in animal models induced by different TiO_2_ particles.

Structural Feature	Animals	Dose	Exposure Route	Toxicity Effect	Reference
Ultrafine TiO_2_ (~20 nm), Fine TiO_2_ particles (~250 nm)	Rats	23.5 and 22.3 mg/m^3^	Intratracheal instillation for 6 h per day, 5 day per week for 12 weeks	Ultrafine particles at equivalent masses access the pulmonary interstitium to a larger extent than fine particles; pulmonary clearance of ultrafine particles was slower (*t*½ = 501 days) than of larger particles (*t*½ = 174 days); a similar mass deposition of the two particle types in the lower respiratory tract; ultrafine particles elicited a persistently high inflammatory reaction compared to the larger-sized particles; this correlated well with their greater surface area per mass.	[10,34]
Ultrafine TiO_2_ (~20 nm), larger TiO_2_ particles (less than 200 nm)	Rats		Intratracheal instillation	Ultrafine particles highly access the pulmonary interstitium; PMNs influx into the alveolar space; The acute inflammatory reaction including an increased percentage of neutrophils, γ-glutamyl transpeptidase concentration (a measure of cell damage), protein concentration (a measure of epithelium permeability), and lactate dehydrogenase (LDH) in BALF were induced.	[20]
Rutile nano-TiO_2_ (21 nm)	Mice	0.1, and 0.5 mg	Intratracheal instillation for one time	Pulmonary emphysema, extensive disruption of alveolar septa, type II pneumocyte hyperplasia, epithelial cell apoptosis, and accumulation of particle-laden macrophages were induced.	[36]
Fine TiO_2_ (250 nm mean diameter)	Mice		Intratracheal instillation for 4, 24, or 72 h	Inflammatory cells or expression of inflammatory cytokines were not detected in the lung tissue.	[37]
Ultrafine TiO_2_ particles (1.4 μm)	Mice, Rats, Hamster	10, 50, and 250 mg/m^3^	Inhalation for 6 h per day, 5 day per week for 12 weeks	Species differences in pulmonary responses: rats developed a more severe and persistent pulmonary inflammatory response than either mice and hamsters; hamsters are better able to clear TiO_2_ NPs than similarly exposed mice and rats.	[17,18]
Anatase TiO_2_ nanospheres, short belts (1–5 μm), long nanobelts (4–12 μm)	Mice	0–30 μg	Pharyngeal aspiration	Both nanospheres and long nanobelts resulted in the lung deposition of 135 μg TiO_2_. At 112 day after exposure, the lung burden was significantly lower in nanosphere-exposed mice than in nanobelt-exposed mice.	[39]
Rutile TiO_2_ nanorods	Wistar Rats	1, and 5 mg/kg	Intratracheal instillation for 24 h	Inflammation responses were examined in BALF (significantly increased neutrophilic inflammation) and whole blood (significantly reduced platelets and elevated numbers of monocytes and granulocytes) at doses of 1 or 5 mg/kg.	[40]
Nanoscale TiO_2_ rods (anatase = 200 nm × 35 nm), nanoscale TiO_2_ dots (anatase = similar to 10 nm)	Rats	1 and 5 mg/kg	Intratracheal instillation	Produced transient lung inflammation and cell injury in rats at 24 h post-exposure, which is similar to the pulmonary effects of rutile TiO_2_ NPs (300 nm).	[41]
Anatase/rutile spheres (TiO_2_-P25), anatase spheres (TiO_2_-A), anatase nanobelts (TiO_2_-NBs)	Mice and Rats	20, 70, and 200 μg	Intratracheal instillation	TiO_2_-A, TiO_2_-P25, and TiO_2_-NB caused significant neutrophilia in mice at 1 day in three of four labs, and this effect was resolved by day 7; TiO_2_-P25 and TiO_2_-A had no significant effect in rats in any of the labs; Only TiO_2_ nanobelts caused significant neutrophilia in rats at 1 day after intratracheal instillation in two or three of four labs.	[42,43]
Base TiO_2_ particles, TiO_2_ particles coated with aluminum oxide (0%–6%) and/or silica (0%–11%)	Rats	2 and 10 mg/kg; 1130–1300 mg/m^3^ (high dose)	Intratracheal inhalation and instillation for 4 weeks	Surface-coated TiO_2_ produced higher pulmonary inflammation (PMNs in BALF) than the uncoated TiO_2_ at 24 h in SD rats, but this effect was only a short-term, transient lung inflammatory response and was reversible at one week post-exposure; Surface treatments influenced the toxicity of TiO_2_ particles.	[44]
In situ-produced TiO_2_ (~21 nm), rutile (<5 μm), nanosized rutile/anatase (~30 nm), nanosized anatase (<25 nm), silica-coated nanosized needle-like rutile (~10 × 40 nm)	Mice	10 mg/m^3^	Inhalation for 2 h, 4 consecutive days, 4 weeks	Only SiO_2_-coated rutile commercial TiO_2_ NPs elicited clear-cut pulmonary neutrophilia, increased expression of tumor necrosis factor (TNF)-α and neutrophil-attracting chemokines; The level of lung inflammation could not be explained by the surface area of the particles, their primary or agglomerate particle size, or free radical formation capacity but was rather explained by the surface coating.	[45]
Hydrophobic and silanized ultrafine TiO_2_	Rats	250 and 500 μg	Intratracheal instillation	Silanized TiO_2_ did not show toxicity, but a much lower pulmonary inflammation was induced in comparison to the hydrophilic uncoated TiO_2_ in rat lung; Surface properties (surface chemistry) appeared to play an important role in ultrafine particle toxicity.	[46]
Pristine TiO_2_ NPs, TiO_2_ NPs embedded in paints	Mice	20 μg	Oropharyngeally aspiration once a week for 5 weeks	The paint containing TiO_2_ ENPs did not modify macrophage and neutrophil counts, but mildly induced KC and IL-1β; The incorporation of TiO_2_ NPs in aged paint matrix blocked most of the particle-induced lung and systemic blood toxicity.	[47]
Rutile TiO_2_ NPs coated with alumina (uf-1), rutile TiO_2_ NPs coated silica/alumina (uf-2), uncoated anatase/rutile TiO_2_ (uf-3)	Rats	1 or 5 mg/kg	Intratracheal instillation	uf-1 and uf-2 produced transient lung inflammation, and uf-3 produced pulmonary inflammation, cytotoxicity and adverse lung effects, and aggregated macrophages in the alveolar regions of the lung; uf-3 particles showed more chemical reactivity than both uf-1 and uf-2 particles.	[48]
Surface-coated rutile TiO_2_ (~20.6 nm) (coating content: silicon, aluminum, zirconium and polyalcohol)	Mice	18, 54, and 162 μg	Intratracheal instillation for one time	Nano-TiO_2_ deposited in the lung; 3000 genes were altered in the pulmonary system; At low doses, surface-coated rutile TiO_2_ potentially down-regulated several gene expression associated with ion homeostasis and muscle function in the absence of inflammation.	[27]
Commercially TiO_2_ P25 untreated with hydrophilic surface, TiO_2_ T805 silanized with hydrophobic surface	Rats	0.15, 0.3, 0.6 and 1.2 mg	Instillation for one time	There was no inflammation or persistent DNA damage in the lung of rats exposed to two types of commercial TiO_2_ at low doses administered.	[49]
Fine (180 nm) and ultrafine (20–30 nm) TiO_2_ particles (hydrophilic), surface modified with methylation (hydrophobic)	Rats	1 and 6 mg	Intratracheal instillation for 16 h	A lesser inflammatory response (influx of neutrophils, activated PMNs and total cell number) was induced in rats in comparison to the untreated TiO_2_; the impact of surface methylation on TiO_2_ toxicity was negligible; surface area rather than hydrophobic surface determined the pulmonary inflammation.	[50]

**Table 2:** Cytotoxicity of TiO_2_ particles with different structural features.

Structural Feature	Cell Line	Dose and Exposure Time	Cytotoxicity Effect	Reference
Ultrafine TiO_2_ (29 nm mean diameter, 50 m^2^/g surface area), fine TiO_2_ (250 nm mean diameter, 6.6 m^2^/g surface area)	Macphage cell line (J774.2)	125.45 mg/mL for 4, 8, 24, and 48 h	Ultrafine and fine particles had no significant cytotoxic effects on J774.2 AM ultrafine TiO_2_ significantly impair the ability of J774.2 mouse AM to phagocytose 2 μm indicator latex beads more than the fine TiO_2_.	[33]
27 nm TiO_2_ particles	Human bronchial epithelial cells (BEAS 2B)		27 nm TiO_2_ was internalized into BEAS-2B cells and proximity to cellular nuclei between 5 min and 2 h.	[35]
Nanosized TiO_2_ particles (10 and 20, 200 nm)	BEAS 2B		Nanosized TiO_2_ particles (10 and 20 nm) induced the oxidative DNA damage, lipid peroxidation, and micronuclei formation in the absence of light, but larger sized TiO_2_ (>200 nm) did not induce any oxidative stress and DNA damaging events; rutile-sized 200 nm particles induced hydrogen peroxide and oxidative DNA damage in the absence of light but the anatase-sized 200 nm particles did not.	[38]
Spherical TiO_2_ NPs (12–140 nm; both anatase and rutile)	Human lung carcinoma epithelial cell line (A549 cells)		Single strand breaks, oxidative lesions to DNA and oxidative stress were induced; the cells ability to repair DNA was impaired.	[51,52]
TiO_2_-based nanofilaments	Human lung tumor cells (H596)	0.01, 0.1, 1, and 2 μg/mL	TiO_2_-based nanofilaments (2 μg/mL) impaired cell proliferation and cell death in a dose-dependent manner; The short (<5 μm) needle-like structures were taken up by H596 cells and clustered and gathered around the cell nucleus.	[53]
TiO_2_ nanobelts: short (<5 μm) long (>15 μm)	Primary murine alveolar macrophages	100 μg/mL	The 15-μm nanobelts were highly toxic, involving the loss of lysosomal integrity and the release of cathepsin B. These fiber-shaped nanomaterials induced inflammasome activation and the release of inflammatory cytokines in a manner very similar to asbestos or silica.	[54]
0-D TiO_2_ nanoparticles, 1-D TiO_2_ nanorods, 3-D TiO_2_ assemblies	HeLa cells	125 μg/mL	0-D anatase NPs decreased cell viability to a level of 80% at 125 μg/mL, and cell viability of 1-D and 3-D structures remained close to 100%; 0-D TiO_2_ NPs and 1-D nanorods could be readily internalized into the cells and the spherical particles were taken up more than the rod-shaped particles of similar size; 3-D assembled aggregates of TiO_2_ were less likely to be incorporated into cells.	[55]
Anatase/rutile spheres (TiO_2_-P25), anatase spheres (TiO_2_-A), anatase nanobelts (TiO_2_-NBs)	Human monocyte/macrophage cell line (THP-1)	10, 25, 50, and 100 μg/mL for 24 h	TiO_2_ was not cytotoxic except for the nanobelt form, which was cytotoxic and induced significant IL-1β production in THP-1 cells.	[56]
Anatase and rutile TiO_2_ NPs	A549		Anatase TiO_2_ produced greater cell responses and was more toxic than rutile by MTT and XTT assay. Differences in biological response of NPs occurred as a function of size, crystalline phase and chemical composition.	[57]
Nanocrystalline TiO_2_ (anatase and rutile)	A549 and human dermal fibroblasts (HDF) cell line	100 μg/mL	Anatase was 2 orders of magnitude more cytotoxic (LC_50_ of 3.6 µg/mL) than similarly sized rutile counterparts (LC_50_ of 550 µg/mL) by determining cell viability and LDH release; The most cytotoxic NPs were the most effective for generating ROS, and were more likely to generate damaging RS species in cell culture.	[58]
Nanosized anatase (<25 nm), nano-sized rutile with SiO_2_ coating, and fine rutile (<5 µm)	BEAS-2B, Chinese hamster lung fibroblast (V79) cells	1–100 μg/cm^2^ for 24, 48, and 72 h	Nano-sized anatase and fine rutile induced DNA damage at doses of 1 and 10 μg/cm^2^, while SiO_2_-coated rutile induced DNA damage only at 100 μg/cm^2^. Only nanosized anatase could elevate the frequency of micronucleated BEAS-2B cells.	[59,60]
Anatase and rutile TiO_2_ NPs (6.3, 10, 50, and 100 nm)	Mouse keratinocyte cell line (HEL-30)	0, 10, 25, 50, 100, and 150 μg/mL for 24 h	Anatase TiO_2_ NPs could induce cell necrosis, whereas rutile TiO_2_ NPs could initiate apoptosis through the formation of ROS.	[61]
Uncoated TiO_2_ (anatase and rutile), polyacrylate-coated nano-TiO_2_	Chinese hamster lung fibroblast (V79) cells	10 and 100 mg/L for 24 h	Both coated and uncoated TiO_2_ (anatase and rutile) decreased the cell viability in a mass- and size-dependent manner; TiO_2_ NPs coated with polyacrylate were only cytotoxic at high concentration (100 mg/L), and only uncoated nano-TiO_2_ induced DNA damage.	[60]
Functionalized TiO_2_ NPs with various surface groups (–OH, −NH_2_, and –COOH)	Lewis lung carcinoma, 3T3 fibroblasts	0.01, 0.1, 1, and 10 mg/L for 24 h	–NH_2_ and –OH groups showed significantly higher toxicity than –COOH; the decreased cell viability was associated with TiO_2_ particles-induced protein aggregation/denaturation and subsequent impaired cell membrane function.	[62]
Rutile (<5 μm), nanosized rutile/anatase (~30 nm), nanosized anatase (<25 nm), silica-coated nanosized needle-like rutile (~10 × 40 nm) (cnTiO_2_)	Murine macrophages RAW 264.7; Human pulmonary fibroblasts (MRC-9)	20, 30, 100, 300 μg/mL for 6 h	cnTiO_2_ elicited significant induction of TNF-α and neutrophil-attracting chemokines. Stimulation of human fibroblasts with cnTiO_2_-activated macrophage supernatant induced high expression of neutrophil-attracting chemokines, CXCL1 and CXCL8.	[45]
Pure anatase and rutile TiO_2_	Human alveolar type-I-like epithelial cell (TTI)		These two nano-TiO_2_ forms mediated a similar profile and pattern of inflammatory response; pure rutile caused a small, but consistently greater response for IL-6, IL-8 and MCP-1; the temporal induction of oxidative stress varied markedly between the two nano-TiO_2_ forms.	[63]

At the nano level, TiO_2_ NPs at a given size can agglomerate in different sizes and structures. Influence of the agglomeration of TiO_2_ NPs on pulmonary toxicity has been investigated by Noël *et al.* [64,65,66]. One study reported that rats exposed to small agglomerates (<100 nm) of 5 nm TiO_2_ by inhalation showed greater cytotoxic and oxidative stress responses than rats exposed to larger agglomerates (>100 nm) of the same NPs, which only induced a slight inflammatory reaction [64]. In a follow-up study, the authors compared the agglomeration state with different primary sizes of TiO_2_ NPs (5, 10–30 and 50 nm) [66]. The results showed that for an agglomeration state smaller than 100 nm, the 5-nm particles caused a significant increase of LDH activity (cytotoxic effects) compared to controls, while oxidative damage measured by 8-isoprostane concentration was less when compared to 10-, 30- and 50-nm particles. This indicates that the initial particle size and agglomeration state of TiO_2_ NPs are important factors for the lung inflammatory reaction and cytotoxic and oxidative stress responses.

### 2.2. Shape

TiO_2_ NPs or nanospheres are generally considered to have cytotoxicity effects, which have been thoroughly assessed and published. Jugan *et al.* [51,52] reported that spherical TiO_2_ NPs (12–140 nm; both anatase and rutile) induced single strand breaks, oxidative lesions to DNA and oxidative stress in A549 cells (human lung carcinoma epithelial cell line). They also showed that TiO_2_ NPs impair the cells ability to repair DNA by deactivating both the nucleotide excision repair (NER) and the base excision repair (BER) pathways.

Other than zero-dimensional TiO_2_ NPs (nanospheres), one-dimensional TiO_2_ nanostructures are the most synthesized and widely used including nanorods, nanobelts, and nanotubes, *etc*. The shape of TiO_2_ NPs has an effect on their deposition in the lung. The exposure of mice to various shapes of anatase TiO_2_ (nanospheres, short belts of 1–5 μm, and long nanobelts of 4–12 μm) resulted in the lung deposition of 135 μg for the animals exposed to both nanospheres and long nanobelts. At 112 day after exposure, the lung burden was significantly lower in nanosphere-exposed mice than in nanobelt-exposed mice [39]. Several works report the interaction of NP shape with lung tissue or pulmonary cells. In a study of rutile TiO_2_ nanorods, inflammation responses were examined in BALF (significantly increased neutrophilic inflammation) and whole blood (significantly reduced platelets and elevated numbers of monocytes and granulocytes) in Wistar rats 24 h after intratracheal instillation at doses of 1 or 5 mg/kg [40]. Warheit *et al.* [41] showed that instilled nanoscale TiO_2_ rods (anatase = 200 nm × 35 nm) and nanoscale TiO_2_ dots (anatase = similar to 10 nm) produced transient lung inflammation and cell injury in rats at 24 h post-exposure, which is similar to the pulmonary effects of rutile TiO_2_ NPs (300 nm). The cytotoxic effect of TiO_2_-based nanofilaments on H596 human lung tumor cells have been evaluated [53]. The addition of TiO_2_-based nanofilaments (2 μg/mL) impaired cell proliferation and cell death in a dose-dependent manner. The short (<5 μm) needle-like structures were taken up by H596 cells and clustered around the cell nucleus. Hamilton *et al.* [54] synthesized the short (<5 μm) and long (>15 μm) TiO_2_ nanobelts and tested their biological activity using primary murine alveolar macrophages and mice. The 15-μm nanobelts were highly toxic, involving the loss of lysosomal integrity and the release of cathepsin B. These fiber-shaped nanomaterials induced the inflammasome activation and the release of inflammatory cytokines in a manner very similar to asbestos or silica. At the lowest-observed-effect level (LOEL), only the anatase TiO_2_ nanobelt displayed significant inflammation in the BALF of Sprague Dawley (SD) rats 1 day after intratracheal instillation compared with anatase/rutile P25 spheres (TiO_2_-P25) and pure anatase spheres [42]. The various morphological classes of TiO_2_ nanostructures, including zero-, one-, and three-dimensional (0-D, 1-D, and 3-D) anatase assemblies, have been evaluated [55]. At a concentration of 125 μg/mL, 0-D anatase NPs decreased cell viability to a level of 80%, and the cell viability of 1-D and 3-D structures remained close to 100%. The cellular uptake experiment showed that 1-D nanorods and 0-D TiO_2_ NPs could be readily internalized into the cells after 24 h incubation. The spherical particles were taken up more than the rod-shaped particles of similar size. The more sterically unwieldy, highest surface area 3-D aggregates of TiO_2_ were less likely to be incorporated into cells. The National Institute of Environmental Health Science (NIEHS) Nano GO Consortium conducted a series of coordinated inter-laboratory research with different shapes of TiO_2_ both *in vitro* and *in vivo*. The results showed that only the TiO_2_ nanobelt form was toxic; it induced significant IL-1β production in THP-1 (human monocyte/macrophage cell line) cells, and caused significant neutrophilia in mice and rats at 1 day after intratracheal instillation in two or three of four labs. However, no significant toxicity effect was observed *in vitro* for TiO_2_ spheres in any of the labs [43,56].

### 2.3. Crystal Phase

Anatase and rutile have different crystal lattices. Rutile is considered as an inert form, whereas anatase is an active form of TiO_2_ with a high refractive index and low scattering and strong absorption of ultraviolet (UV) radiation. Based on crystal structure as the mediating property, nanotoxicity studies examining the effects of TiO_2_ have shown the induction of inflammatory responses, cytotoxicity and reactive oxygen species (ROS) formation. Ferin *et al.* [67] exposed rats to an aerosol of either anatase or rutile by intratracheal instillation in doses of 0.5 or 5.0 mg/rat and determined the TiO_2_ retention in the lung up to 132 days post-exposure. They found that both anatase and rutile TiO_2_ yielded similar results in lung response such as AM, peroxidase positive AM, and PMN leukocytes. Although both anatase and rutile TiO_2_ NPs could be taken up into cells, located in the cytoplasm, and isolated in vacuoles, the cytotoxicity of NPs depends, to some extent, on crystalline structure. Anatase TiO_2_ NPs are found to produce greater cell responses and to be more toxic than rutile TiO_2_ by MTT and XTT assay [57]. Using the A549 and HDF cell lines, Sayes *et al*. demonstrated that catalytically active anatase was 2 orders of magnitude more cytotoxic (LC_50_ of 3.6 µg/mL) than its similarly sized rutile counterpart (LC_50_ of 550 µg/mL) by determining cell viability and LDH release [58]. The profile and pattern of inflammatory mediator release and temporal induction of oxidative stress were determined using TiO_2_ NPs synthesized specifically for toxicological study and a highly relevant human lung cell model, The authors showed that it was a useful approach to delineating the physiochemical properties of nanomaterials in cellular reactivity [63].

The genotoxicity of nanosized anatase (<25 nm), nanosized rutile with SiO_2_ coating, and fine rutile (<5 μm) in human BEAS-2B cells was assessed by Falck *et al.* [59]. Nanosized anatase and fine rutile induced DNA damage at the doses of 1 and 10 μg/cm^2^, while SiO_2_-coated rutile induced DNA damage only at 100 μg/cm^2^. Only nanosized anatase could elevate the frequency of micronucleated BEAS-2B cells. Another study showed a similar result. The anatase nano-TiO_2_ caused a stronger induction of DNA damage than rutile in Chinese hamster lung fibroblast (V79) cells as determined by comet assay [60].

In addition, there are several papers about the cytotoxicity of rutile and anatase TiO_2_ NPs in other cell lines. Braydich-Stolle *et al.* [61] reported that anatase TiO_2_ NPs, regardless of size, could induce cell necrosis, whereas rutile TiO_2_ NPs could initiate apoptosis through the formation of ROS. The correlation between crystal phase and oxidant capacity was established using TiO_2_ NPs of 11 different crystal phase combinations at similar sizes [68]. The ability of anatase TiO_2_ NPs to generate ROS was higher than anatase/rutile mixtures and rutile samples.

### 2.4. Surface Coating

In commercial applications of TiO_2_, surface coating with inorganic or organic substances is often used to facilitate dispersion, solubility, UV protection, wearing and plastics. The toxicity of coated TiO_2_ has been evaluated by many researchers, and it is well known that nanomaterial interactions with biology are dictated by the chemical functionalities on the surface in addition to their shape and size. Hydrophobic ultrafine TiO_2_ coated with a silane compound was initially reported to be highly toxic and lethal when administrated to rats by intratracheal injection [69]. Since then, the surface coating of TiO_2_ with aluminum oxide and/or silica has been shown to produce higher pulmonary inflammation (PMNs in BALF) than the uncoated TiO_2_ at 24 h in SD rats administered a large dose of 10 mg/kg [44], but this effect was only a short-term, transient lung inflammatory response and was reversible at one week post-exposure. A similar result was observed with TiO_2_ particles having a silane coating (hydrophobic) compared to uncoated TiO_2_ (hydrophilic). Rossi *et al.* [45] showed that only SiO_2_-coated rutile commercial TiO_2_ NPs elicited clear-cut pulmonary neutrophilia, increased expression of tumor necrosis factor (TNF)-α and neutrophil-attracting chemokines both *in vivo* (BALB/c mice, by inhalation at 10 mg/m^3^) and *in vitro* (RAW264.7 and human pulmonary fibroblasts MRC-9 cells). They concluded that the level of lung inflammation could not be explained by the surface area of the particles, their primary or agglomerate particle size, or free radical formation capacity but rather by the surface coating. Oberdorster *et al.* reported, however, that 500 μg hydrophobic and silanized ultrafine TiO_2_ did not show toxicity, but a much lower pulmonary inflammation was induced in comparison to the hydrophilic uncoated TiO_2_ in rat lung [46]. The incorporation of TiO_2_ NPs in aged paint matrix blocked most of the particle-induced lung and systemic blood toxicity in BALB/c mice [47]. The rutile TiO_2_ NPs coated with alumina (uf-1) and silica/alumina (uf-2) produced transient lung inflammation in rats exposed by intratracheal instillation at doses of 1 or 5 mg/kg, and uncoated anatase/rutile TiO_2_ (uf-3) induced cytotoxicity and aggregated macrophages in the alveolar regions of the lung. This occurred because uf-3 particles showed more chemical reactivity than both uf-1 and uf-2 particles [48]. At low doses, surface-coated rutile TiO_2_ deposited in the mice lung may potentially perturb several gene expression associated with ion homeostasis and muscle function in the absence of inflammation [27].

The cytotoxicity and genotoxicity of coated and uncoated TiO_2_ particles were recently reported using Chinese hamster lung fibroblast (V79) cells [60]. The authors found that both coated and uncoated TiO_2_ (anatase and rutile) decreased the cell viability in a mass- and size-dependent manner, although the TiO_2_ NPs coated with polyacrylate were only cytotoxic at high concentration (100 mg/L), and only uncoated nano-TiO_2_ induced DNA damage. Rehn *et al.* [49] determined that there was no inflammation or persistent DNA damage in the lung of rats exposed to two types of commercial TiO_2_ (untreated with hydrophilic surface and silanized with a hydrophobic surface) at low doses (a single dose of 0.15, 0.3, 0.6 and 1.2 mg) by instillation. Methylated TiO_2_ particles induced a lesser inflammatory response (influx of neutrophils and total cell number) in rats after intratracheal instillation in comparison to the untreated TiO_2_, and the impact of surface methylation on TiO_2_ toxicity was negligible [50]. However, the question remains: what is the underlying mechanism? Thevenot *et al.* tested the cytotoxicity effect of functionalized TiO_2_ NPs with various surface groups (–OH, –NH_2_, and –COOH) and reported that the decreased viability of TiO_2_ NPs on lung epithelial cells was associated with TiO_2_ particles-induced protein aggregation/denaturation and subsequent impaired cell membrane function [62]. Thus, it can be seen that surface treatment can influence the toxicity of TiO_2_ particles in the lung, and the pulmonary toxicity and cytotoxicity of coated TiO_2_ NPs might be related to surface chemical activity because of different surface coatings [62].

### 2.5. ROS Mechanism in Lung Toxicity of TiO_2_ NPs

Inhaled TiO_2_ NPs show considerably stronger pulmonary inflammatory effects and the mechanism that has been suggested to be involved included ROS production as a hallmark in TiO_2_ NP toxicity [2,5,70,71], especially under exposure to light or UV. Sayes *et al.* [58] detected that photoactivated TiO_2_ produced greater ROS and resulted in cytotoxicity. This effect is better described by the crystal structure and the specific surface area than mass dose.

Anatase TiO_2_ NPs are capable of reacting with a wide range of organic and biological molecules and are more prone to generate ROS than the rutile form. At the cellular level, ROS may be generated directly by particle structures in or near the cell or may arise more indirectly due to the effects of internalized particles on mitochondrial respiration or the depletion of antioxidant species within the cell. In an *in vitro* cell assay, Bhattacharya *et al.* [72] reported that anatase TiO_2_ particles with diameters <100 nm were able to generate elevated amounts of free radicals and induced DNA-adduct formation (8-OHdG) but not DNA-breakage after uptake by human lung fibroblasts (IMR-90) and BEAS-2B. The anatase TiO_2_ NPs (50, 100, 200 and 300 μg/mL) induced dose-dependent mitochondrial injury and ATP synthesis prevention in A549 cells owing to the over-generation of ROS [73]. The radical generation driven by the surface area of TiO_2_ NPs in A549 cells was investigated by Singh *et al.* [74]. They observed that the commercial TiO_2_ NPs elicited significant increased ROS generation during cell treatment and indicated that the higher specific surface area of particles caused oxidative stress in A549 cells rather than hydrophobicity [74]. In a rapid cell-free pre-screening assay, Jiang *et al.* [68] investigated the role of crystal structure and surface area on particle ROS generation and established that size, surface area, and crystal structure all contribute to ROS generation. ROS generation was associated with the number of defect sites per surface area, and an S-shaped curve was observed as a function of particle size. The ability of TiO_2_ NPs to generate ROS was amorphous > anatase > anatase/rutile mixtures > rutile.

ROS are also produced by lung AM and inflammatory cells during overloading and immunological responses of the lung to inhaled TiO_2_ NPs. When antioxidant defenses are overwhelmed, oxidative stress can occur and is considered an underlying mechanism of the proliferative and genotoxic responses to inhaled TiO_2_ NPs. Sun *et al.* [75] investigated that TiO_2_ NPs significantly accumulated and increased ROS production (elevated O_2_^−^, H_2_O_2_) in mouse lung by intratracheal administration with increasing exposure term. The exposure of human and rodents AM to TiO_2_ NPs caused extracellular ROS generation, resulted in increased TNF-α release, heme oxygenase (HO)-1 mRNA and inducible nitric oxide synthase (iNOS) mRNA expression, and induced an increase in the expression of nuclear factor erythroid 2 related factor 2 (Nrf 2) to adapt intracellular responses to TiO_2_-induced oxidative stress [45,76,77,78]. Xia *et al.* [77] concluded that the surface properties of NPs and their interactions with cellular components were capable of generating oxidative stress.

## 3. Conclusions

Nanoparticles generate great benefits, as well as some potential risks, to human health. Owing to the small size, coupled with the unique physical and chemical properties of NPs, nanotoxicology is put forward by some pioneer scientists to specifically address the problems likely to be caused by nanoparticles/nanomaterials in terms of their potential adverse health effects. In bulk, TiO_2_ is considered to be low toxicity and is widely used in many fields. However, at nanoscale, TiO_2_ can deposit in the alveolar region and access the lung interstitium after inhalation exposure, eliciting a pulmonary inflammatory response and lung injury.

In this paper, an overview of the lung injury and cytotoxicity of TiO_2_ NPs correlated with the physicochemical properties, including size, shape, crystal structure and surface coating is presented. Mass dose is traditionally viewed as the key factor in toxicity studies. However, the biological activity of NPs strongly depends on physicochemical parameters but not on routinely considered in toxicity screening. The unique characteristics of NPs are predominantly associated with their nanoscale structure, size, shape and structure-dependent electronic configurations and an extremely large surface-to-volume ratio relative to bulk materials. The nano size with a high aspect ratio (nanorod, nanobelt, nanofilament) determines the high reactivity of NPs, which enables the insoluble TiO_2_ NPs to agglomerate and affect cellular uptake and lung injury. Because of the different crystal lattices, anatase TiO_2_ induces greater ROS production and cell responses and is more toxic than rutile due to the active sites on its surface. The coating of TiO_2_ with silica and alumina can reduce the pulmonary inflammatory response and cytotoxicity to a certain extent. Lastly, the correlation of ROS production in pulmonary toxicity with properties of TiO_2_ NPs was briefly described.

Nanotoxicology and the biological effects of NPs have become hot topics in many fields. When evaluating the potential biological effects of NPs and elucidating their mechanisms for toxicity screening, the size, crystal phase, dispersion and agglomeration status, coating, and chemical composition should be most appropriately characterized.
